# Variability of Water-Soluble Forms of Choline Concentrations in Human Milk during Storage, after Pasteurization, and among Women

**DOI:** 10.3390/nu11123024

**Published:** 2019-12-11

**Authors:** Sara Moukarzel, Alejandra M. Wiedeman, Lynda S. Soberanes, Roger A. Dyer, Sheila M. Innis, Yvonne Lamers

**Affiliations:** 1 Larsson-Rosenquist Foundation Mother-Milk-Infant Center of Research Excellence, University of California, San Diego, CA 92093, USA; smoukarzel@ucsd.edu; 2 British Columbia Children’s Hospital Research Institute, Vancouver, BC V5Z 4H4, Canada; awiedeman@bcchr.ca (A.M.W.); lynda.soberanes@gmail.com (L.S.S.); radyer@mail.ubc.ca (R.A.D.); sheila.innis@ubc.ca (S.M.I.); 3 Department of Pediatrics, Faculty of Medicine, The University of British Columbia, Vancouver, BC V5Z 4H4, Canada; 4 Food, Nutrition, and Health Program, Faculty of Land and Food Systems, The University of British Columbia, Vancouver, BC V6T 1Z4, Canada

**Keywords:** human milk, donor milk, choline, phosphocholine, storage, pasteurization, milk banking, pumping, breastfeeding, lactation

## Abstract

Choline is critical for infant development and mother’s milk is the sole source of choline for fully breastfed infants until six months of age. Human milk choline consists to 85% of water-soluble forms of choline including free choline (FC), phosphocholine (PhosC), and glycerophosphocholine (GPC). Donor milk requires safe handling procedures such as cold storage and pasteurization. However, the stability of water-soluble forms of choline during these processes is not known. The objectives of this research were to determine the effect of storage and pasteurization on milk choline concentration, and the diurnal intra- and inter-individual variability of water-soluble choline forms. Milk samples were collected from healthy women who were fully breastfeeding a full-term, singleton infant <6 months. Milk total water-soluble forms of choline, PhosC, and GPC concentrations did not change during storage at room temperature for up to 4 h. Individual and total water-soluble forms of choline concentrations did not change after storage for 24 h in the refrigerator or for up to one week in the household freezer. Holder pasteurization decreased PhosC and GPC, and thereby total water-soluble choline form concentrations by <5%. We did not observe diurnal variations in PhosC and total water-soluble forms of choline concentrations, but significant differences in FC and GPC concentrations across five sampling time points throughout one day. In conclusion, these outcomes contribute new knowledge for the derivation of evidence-informed guidelines for the handling and storage of expressed human milk as well as the development of optimized milk collection and storage protocols for research studies.

## 1. Introduction

Choline is an essential nutrient with crucial roles in brain function, neurodevelopment, and growth [1,2]. Biological roles of choline include neurogenesis and synapse formation in the form of acetylcholine, membrane biogenesis, cell division, lipid transports, and myelination in the form of choline phospholipids, and as a methyl donor in the form of its oxidation product betaine [3,4,5,6]. Betaine contributes to the generation of *S*-adenosylmethionine, which is the main methyl donor involved in creatine and phosphatidylcholine synthesis, and DNA methylation, among other biochemical reactions [7]. Choline adequacy in early infancy and rapid stages of growth is critical to support membrane formation, cell proliferation, and parenchymal growth [6].

Exclusive breastfeeding ad libitum is the recommended feeding practice for the first six months of life, with continued breastfeeding up to two years of age [8]. Human milk contains various forms of the essential nutrient choline. The three water-soluble forms of choline, i.e., free choline (FC), phosphocholine (PhosC), and glycerophosphocholine (GPC), contribute to approximately 85% of total choline in human milk; the lipid-soluble phosphatidylcholine and sphingomyelin account for the remaining 15% [9,10,11,12,13].

The practice of human milk pumping and storage for later use is on the rise both at home and in clinical settings to accommodate various situations when feeding human milk at the breast is not possible, e.g., mothers returning to work; insufficient volume of mother’s own milk, and need for donor milk [14,15]. Current guidelines for the safe handling of expressed human milk focus largely on microbiological safety, whereby Holder pasteurization (at 62.5 °C for 30 min) of donor milk is mandatory in all North American hospitals [16]. Additionally, it is recommended that milk be stored in the refrigerator at ≤4 °C for no more than eight days, and in the freezer at −17 °C for up to 12 months [17]. Whilst evidence suggests that refrigeration, freezing and pasteurization impact human milk concentration of folate, vitamin B6, vitamin C, and other nutrients to various degrees [18,19,20,21], the effect of pasteurization and short- and long-term storage at different temperatures on water-soluble forms of choline in expressed human milk is not known.

Water-soluble forms of choline concentrations in human milk may vary within and between women. Higher maternal dietary choline intake increases milk concentrations of water-soluble, yet not fat-soluble, forms of choline, as shown in a 12-week dose-response feeding study and a supplementation trial from 18 gestational weeks to 90 days postpartum [11,13]. No consistent changes in total concentration of water-soluble forms of choline in human milk were observed in six women over a time period of 72 h [22]. The diurnal changes and variability of individual water-soluble choline forms among women have not been studied to date. Understanding these variabilities has important implications for designing study protocols (e.g., sampling techniques) that focus on identifying the determinants of choline in human milk and infant outcomes due to milk choline consumption.

The objectives of this research were to determine the effect of cold storage and pasteurization on the concentrations of water-soluble forms of choline in human milk, and to determine the intra- and inter-individual variability of water-soluble forms of choline concentrations in human milk within a day. Water-soluble forms of choline seem stable during short-term cold storage and most forms remained unchanged after four hours of storage at room temperature and six months of storage at ultra-low freezing temperatures, as employed in research settings. Holder pasteurization significantly impacted milk concentrations of water-soluble forms of choline, but to a small extent that is outweighed by the microbiological safety benefits of pasteurization. We observed intra-individual variability of individual but not total water-soluble forms of choline throughout one day, and recommend standardized sampling protocols for research studies.

## 2. Materials and Methods

### 2.1. Participants and Study Design

The research consisted of two cross-sectional studies, i.e., the stability and variability study, as well as the secondary analysis of bio-banked milk samples for the pasteurization study. For all studies, healthy women who were exclusively breastfeeding a healthy, full-term and singleton infant <6 months of age were eligible to participate. Exclusion criteria included: suffering from diabetes mellitus Type 1 or 2, or any chronic disease involving fat metabolism, taking routine medications known to influence fat metabolism, or consumption of more than 1 alcoholic drink per day.

#### 2.1.1. Stability Study

A convenience sample of 6 mothers of 2- to 6-month old infants was included in this study. Women were recruited through active and passive recruitment methods in the Greater Vancouver area in 2014. Signed informed consent was obtained prior to enrolment. Information on maternal and infant age, sociodemographic status, supplement use, and general infant health, was collected using a self-administered questionnaire. Ethical approval was granted by the University of British Columbia and the British Columbia Children’s and Women’s (C&W) Hospital Research Ethics Board (H12-03191).

Women provided a fresh complete milk expression collected in the morning (between 9:00–10:00 a.m.) using a commercial pump (Medela); samples were immediately transferred on ice to the lab to be aliquoted and analyzed. One milk aliquot was analyzed immediately, with the rest of the samples being stored at different temperatures for different durations (see Supplemental Table S1), prior to analysis.

#### 2.1.2. Pasteurization Study

Mid-feed human milk samples (milk collected after breastfeeding or pumping milk for approximately 3 min) from mothers of 1-month old infants were used in the pasteurization study; these bio-banked samples were derived from a completed, randomized controlled trial studying the effect of DHA supplementation during pregnancy on the cognitive and visual outcomes on infants [23]. The study was approved by C&W REB (H03-70242). A subset of milk samples was randomly selected from the bio-banked samples that had been stored for 5–9 years at −80 °C. In the original study, milk samples were immediately frozen at home for a maximum time of 3 days, and transferred on ice to the lab where they were frozen at −80 °C until analysis. Original milk samples obtained from the participants were thawed on ice, homogenized by gentle mixing, and divided in two aliquots. The first aliquot was stored at −20 °C for 1–2 h, and the second aliquot underwent Holder pasteurization in a water bath. Water-soluble forms of choline were quantified before and after pasteurization on the same day.

#### 2.1.3. Variability Study

Twenty women were enrolled in this cross-sectional study. Recruitment and enrolment procedures were similar to those described for the stability study. Sociodemographic, health and supplement use data were collected using the same self-administered questionnaire as in the stability study. Ethical approval was granted by the University of British Columbia and the British Columbia Children’s and Women’s Hospital Research Ethics Board (H12-03191).

Each participant provided 5 mid-feed milk samples at different times throughout one day. For feasibility purposes, the five different time points were flexible as follows: before breakfast, before lunch, 45–60 min after lunch, 45–60 min after dinner, and before bedtime. Participants were instructed to place the vials in their home freezers immediately after pumping. Frozen samples were transferred to the lab on ice the day after collection and stored at −80 °C for 2 weeks, when choline analysis was completed.

### 2.2. Human Milk Choline Quantitation

Concentrations of water-soluble forms of choline in milk were determined using isotope dilution liquid chromatography tandem mass spectrometry as previously described [24]. In brief, aliquots of 20 μL of human milk were transferred to Eppendorf tubes containing 10 μL of deuterium-labeled internal standards (choline-d9, PhosC-d9, GPC-d9) and vortexed. Protein was precipitated with 30 μL of methanol with 0.1% formic acid. The supernatant was recovered after centrifugation at 18,000 × *g* at 4 °C for 10 min, transferred to an autosampler vial and mixed with acetonitrile with 0.1% formic acid in dilutions of 1:5. The inter-assay and intra-assay coefficient of variation (CV) based on 5 replicates were as follows: For FC, 5.5% and 4.1%; for PhosC, 6.4% and 5.2%; and for GPC 9.5% and 2.3%; respectively.

### 2.3. Statistical Analysis

Participant characteristics are presented using descriptive statistics. Normality of data distribution was assessed using Shapiro–Wilk test. Differences in milk concentrations of water-soluble forms of choline under different storage conditions were determined using the related-samples Friedman test, followed by Wilcoxon signed-ranks test as post-hoc analysis and adjusted using the Bonferroni correction. Differences in water-soluble forms of choline concentrations after pasteurization were determined using the Wilcoxon signed-ranks test. Intra-individual variability in water-soluble forms of choline concentrations was determined using the related-samples Friedman test, followed by Wilcoxon signed-ranks test as post-hoc analysis and adjusted using the Bonferroni correction. Analyses were performed using the IBM SPSS statistics software (IBM SPSS Statistics for Windows, Version 25.0. SPSS Inc., Chicago, IL, USA). Level of significance was set at *p* values < 0.05.

## 3. Results

### 3.1. Participant Characteristics

The characteristics of women included in each study are summarized in Table 1.

### 3.2. Stability of Water-Soluble Choline Forms at Different Storage Conditions

**Figure 1:**
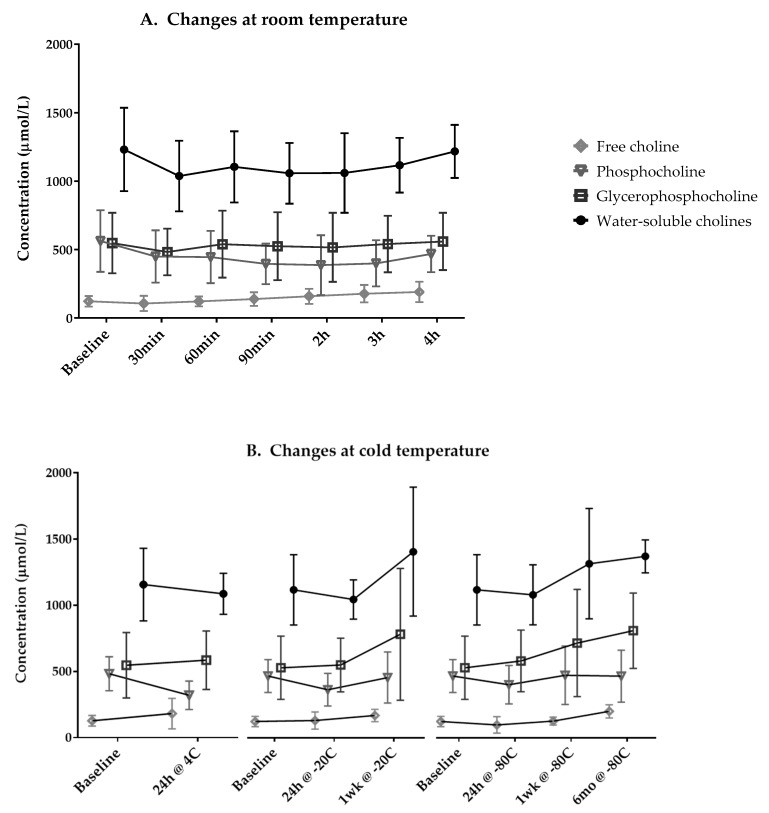
Changes in water-soluble forms of choline concentrations in human milk at (**A**) room temperature (*n* = 6) and (**B**) under different cold storage conditions (*n* = 5). Data presented as mean ± SD.

The concentration of total water-soluble forms of choline, PhosC, and GPC did not significantly change during storage at room temperature for up to 4 h (Figure 1, Supplemental Table S2). Compared to fresh milk samples, i.e., baseline values, only FC concentration significantly increased after 3 h and 4 h of storage at room temperature.

We observed no changes in total water-soluble forms of choline and PhosC concentrations independent of condition and duration of cold storage, including in the refrigerator at 4 °C for 24 h, in the freezer at −20 °C for up to 1 week, and in the ultra-low freezer at −80 °C for up to 6 months (Figure 1, Supplemental Table S2). The concentration of FC and GPC did not change after storage at 4 °C for 24 h or at −20 °C for up to 1 week, but significantly increased between baseline and 6 months of storage at −80 °C.

### 3.3. Stability of Water-Soluble Choline Forms during Pasteurization

Total water-soluble forms of choline concentrations significantly decreased by approximately 5% after Holder pasteurization in 33 milk samples (Table 2). This decrease seems to be largely driven by the decrease in the main water-soluble forms of choline in human milk, i.e., GPC and PhosC. The concentrations of GPC and PhosC showed a mean decrease of 4%–5%. Median FC concentration remained constant and did not seem to be impacted by exposure to pasteurization.

### 3.4. Intra- and Inter-Individual Variability of Water-Soluble Forms of Choline Concentrations

Total water-soluble forms of choline and PhosC concentrations did not vary significantly within a woman during the day based on analysis of five milk samples per mother (Table 3). However, significant changes in FC and GPC were found. Posthoc analysis showed a 22.7% increase in FC from T1 to T4 (*p* = 0.027) and a 12.2% decrease in GPC from T1 to T3 (*p* = 0.027).

**Figure 2:**
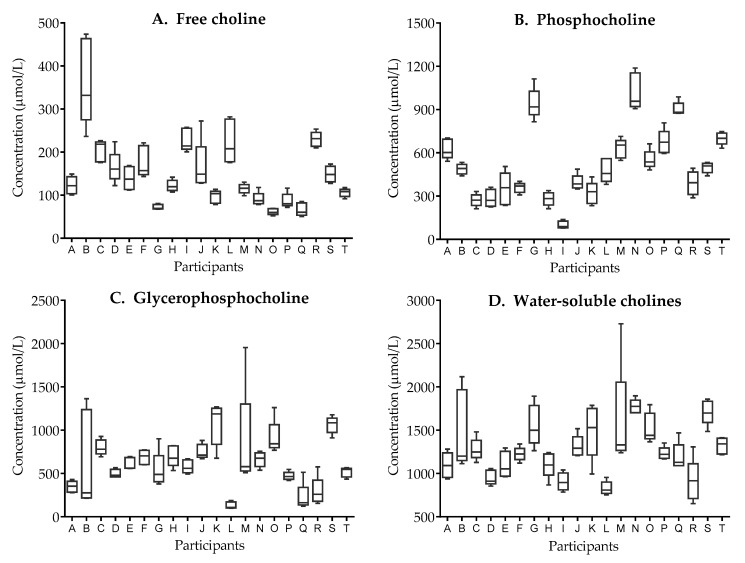
Intra-individual variability of human milk concentration of (**A**) free choline, (**B**) phosphocholine, (**C**) glycerophosphocholine, and (**D**) total water-soluble forms of choline. Boxes represent median and 25^th^–75^th^ percentile; whiskers represent minimum and maximum values. Data for each participant (*n* = 20, represented on the *x*-axis) includes five mid-feed milk samples collected at separate feeds on a single day.

Additionally, as shown in Figure 2, intra-individual variability in FC and GPC concentrations varied between women, whereby milk FC and GPC concentrations showed a large variability in some, i.e., 2 or 3 mothers, with milk samples of most other mothers reflecting minimal variability.

## 4. Discussion

In this study, we report three findings on the stability of water-soluble forms of choline in expressed human milk: First, storing human milk for 3 or 4 h at room temperature significantly increases milk FC concentration. Second, thawing human milk after 6 months of freezing at −80 °C significantly increases FC and GPC concentrations. Third, Holder pasteurization decreases PhosC and GPC concentrations, and thereby lowers total concentration of water-soluble forms of choline. Additionally, we report that total concentration of water-soluble forms of choline does not underlie diurnal variations within a woman; however, significant diurnal variations in FC and GPC concentrations may occur among some, but not all, women. These findings contribute to the literature being harnessed to develop evidence-informed guidelines for the handling and storage of expressed human milk as well as the development of optimized milk collection and storage protocols for research studies.

Milk expression for in-home use or human milk banking have become increasingly recognized as a first best alternative to direct breastfeeding. While ensuring microbiological safety of expressed milk remains a top priority, maintaining milk’s bioactive and nutritional quality is an important weighing factor in developing milk handling and storage guidelines. The impact of storage and pasteurization on the immunological properties, digestive enzymes, antioxidant capacity, and macro- and micronutrient composition in human milk has been recently reviewed [19,25,26,27,28,29], with limited data on water-soluble forms of choline. In an older study, Zeisel et al. [22] compared fresh milk to either samples incubated for 15 min at 37 °C or to samples frozen for 72 h at −10 °C followed by incubation at 37 °C for 15 min. Using radioisotope labeled choline compounds, the authors showed that neither of the two milk handling and storage conditions resulted in significant changes in FC concentration. These findings combined with ours suggest that following current recommendation of keeping freshly expressed milk up to 4 h at room temperature should not significantly alter the composition of water-soluble forms of choline, except for minor possible increases in FC. The possible mechanism(s) underlying the increase in FC concentration cannot be determined in our study as designed.

The increase in FC concentration may be explained by the enzymatic breakdown of the lipid-soluble choline compounds phosphatidylcholine and sphingomyelin. The presence of these phospholipids within the milk fat globule membrane, a complex tri-layer of proteins and lipids, poses an analytical challenge for their accurate quantification [30]. In addition to identifying factors influencing the variability of choline forms in human milk in future studies, it is critical to also investigate whether changes in the composition of choline forms in milk, not total choline concentration per se, have functional implications for infant health and development. Choline has a wide array of functions that support infant growth and development. As an essential component of phosphatidylcholine and sphingomyelin, choline is involved in the maintenance of cell membrane structural integrity and signaling pathways, as well as in parenchymal growth, cell proliferation, and membrane formation [3,4,5,6]. Choline, via its oxidized form betaine, also functions as a methyl group donor in the generation of *S*-adenosylmethionine [7]. Additionally, choline is crucial for brain function as precursor of the neurotransmitter acetylcholine [4]. To date, the contribution of different choline forms in human milk to the variety in choline functions in the developing infant is largely unknown and merits further investigation.

The cold storage conditions we tested are relevant to home and clinical use (refrigeration temperature typically around 4 °C and freezing at −20 °C) and to storage conditions in research settings (ultra-low freeze storage at −80 °C). Our findings suggest that short-term storage of milk aliquots in the refrigerator (at 4 °C) for 1 day and in the home freezer (at −20 °C) for up to one week and long-term storage at −80 °C for up to 6 months does not alter the total concentration of water-soluble forms of choline in expressed human milk samples. However, milk FC and GPC concentrations increased in samples that were thawed after six months of ultra-low freeze storage (i.e., at −80 °C). The increase in FC and GPC concentrations after prolonged freezer storage may be explained by the breakdown of lipid-soluble forms of choline, similar to the increase in FC concentration in milk stored at room temperature for 4 h. Similar changes were observed in serum samples that were inappropriately processed and stored, with increasing concentration of total water-soluble choline concentration [31]. We were limited to six participants for the stability study as it was logistically challenging to recruit participants willing to commute to our research site, provide a fresh milk sample, and provide the sample as a complete milk expression. We however compensated in our statistical analysis for this limitation (i.e., possible high variability across samples due to small sample size) by using related-samples analyses rather than independent comparisons across conditions. Future research is warranted to enhance the stability data by testing more milk samples as well as more frequent and longer duration intervals to identify whether and when water-soluble forms of choline concentration or composition may change in different cold storage conditions.

To our knowledge, this study is the first to test the effect of pasteurization on water-soluble forms of choline concentrations in human milk. We showed that Holder pasteurization lowered milk PhosC and GPC concentrations by about 5%, and thereby total water-soluble forms of choline concentration by 3%. Limitation of our study was the use of previously frozen milk samples, for 5–9 years at −80 °C, which is not consistent with clinical practice of pooling human milk and it undergoing pasteurization. We used biobanked milk samples because of low milk volumes collected in the stability and variability study, as well as to reduce participant burden. Additionally, for this preliminary study, we were mainly focusing on the comparison of choline concentrations between before and after pasteurization. We acknowledge that the long-term storage at −80 °C may have affected the milk concentrations of the water-soluble forms of choline; however, the total water-soluble choline concentration in the pre-pasteurized samples was similar to that of the fresh milk samples in the stability study (1241 μmol/L versus 1231 μmol/L, respectively), as well as compared to those reported in the literature [11,24,32]. This seems to reflect that the total water-soluble choline concentration was not affected by the long-term storage at −80 °C and that our findings may contribute to the literature of how human milk is altered by pasteurization.

Considering the crucial benefits of pasteurization for microbiological safety, we evaluate the <5% decrease in milk concentration of water-soluble forms of choline as minor yet recommend the confirmation of our findings in a second and larger study. The impact of pasteurization on lipid-soluble choline compounds should also be investigated in future studies. If confirmed, our finding of a 5% decrease should reassure clinicians that the benefits of providing pasteurized human milk continue to outweigh the risk of nutrient losses. Indeed, despite previous reports of a decrease in several nutrients, including folate, vitamin C and B6, due to pasteurization [18,25,26], the use of pasteurization techniques continues to ensure the biologically-safe provision of human milk at hospital settings around the globe.

In regards to the variability of water-soluble forms of choline concentration in expressed human milk, we observed diurnal changes in milk FC and GPC concentrations, but not in PhosC and total water-soluble choline forms. The total concentration of water-soluble forms of choline in expressed human milk we found was similar to the concentrations previously reported by our team for Canadian lactating women [24,32] and by Fischer et al. for US women [11]. Because no substantial diurnal changes were observed and the total concentration of water-soluble forms of choline in human milk seems stable, we conclude that the time point of milk sample collection in studies on water-soluble forms of choline may not influence the study outcomes. However, because of the diverse intra-individual variability of FC and GPC concentration within a day, we recommend to standardize the time of milk sample collection across study participants. The influencing factors of the intra-individual variability for some of the water-soluble choline forms, when present, are not fully elucidated but may be related to dietary intake of choline and/or genetic variants related to choline absorption, distribution, and metabolism [11]. The effect of acute versus long-term dietary choline intake on milk composition of water-soluble forms of choline merits further investigation.

In conclusion, we provide new information on the stability of water-soluble forms of choline concentration that will help in the development of evidence-based guidelines for the safe handling and storage of expressed milk samples. Because breast milk is the recommended sole source of nutrients, including that of choline, for infants under the age of 6 months, the handling of expressed milk needs to address microbiological safety as well as nutrient-protective needs. Further research is warranted on the effect of acute versus long-term dietary choline intake on the composition of choline forms in human milk as well as the metabolic and functional significance of individual choline forms on infant growth and development.

## Figures and Tables

**Table 1:** Participant characteristics of each study.

Characteristics Total Sample Size	Stability Study (*n* = 6)	Pasteurization Study (*n* = 33)	Variability Study (*n* = 20)
Age, *y*	35 ± 2 ^1^	34 ± 4	32 ± 4
Postpartum, *mo*	4.0 (3.0) ^2^	1	4.5 (3.0)
First-time breastfeeding, *n* (%)	6 (100)	20 (61)	10 (50)
Ethnic background, *n* (%)			
European	2 (33)	26 (79)	11 (55)
Latin American	2 (33)	2 (6)	4 (20)
Middle Eastern	0	3 (9)	3 (15)
First Nation	1 (17)	0	1 (5)
Chinese Asian	1 (17)	2 (6)	1 (5)

^1^ Mean ± SD; ^2^ median (IQR).

**Table 2:** Water-soluble forms of choline concentrations in human milk before and after Holder pasteurization ^1.^

Choline Form	Before, μmol/L	After, μmol/L	Difference, μmol/L	Difference, %	*p*-Value ^2^
*Free choline*					
Mean ± SD	124 ± 60	124 ± 58	0.6 ± 14	1.3 ± 10	0.893
Median (min; max)	117 (47.9; 293)	120 (44.6; 275)	−1 (−29; 46)	−0.5 (−18; 33.0)	
*Phosphocholine*					
Mean ± SD	675 ± 220	632 ± 189	−43 ± 73	−4.7 ± 10	0.003
Median (min; max)	676 (230; 1131)	621 (261; 960)	−41 (−238; 111)	−6.5 (−21; 25)	
*Glycerophosphocholine*					
Mean ± SD	442 ± 181	425 ± 181	−17 ± 43	−3.6 ± 10	0.015
Median (min; max)	381 (243; 904)	387 (211; 994)	-20 (-122; 97)	-5.6 (-21; 26)	
*Total water-soluble choline*					
Mean ± SD	1241 ± 249	1186 ± 201	−56 ± 124	−3.4 ± 10	0.017
Median (min; max)	1229 (789; 1794)	1136 (792; 1566)	−63 (358; 231)	−4.8 (−20; 27)	

^1^*n* = 33 samples; ^2^ Wilcoxon signed-rank test to test for differences between before and after Holder pasteurization.

**Table 3:** Concentrations of water-soluble forms of choline in mid-feed milk samples collected at five different time points within one day ^1.^

Choline Form	T1	T2	T3	T4	T5	*p* ^2^
*Free choline*						
Median (IQR)	119 (73.5)	125 (63.7)	131 (75.4)	146 (119)	132 (81.1)	0.029
Range	51.9–156	60.4–253	69.2–474	59.9–453	505–332	
*Phosphocholine*						
Median (IQR)	490 (328)	488 (270)	441 (359)	457 (330)	482 (335)	0.545
Range	138–906	82.1–1113	115–1120	78.9–1188	88.9–958	
*Glycerophosphocholine*						
Median (IQR)	625 (194)	559 (280)	549 (320)	533 (275)	682 (408)	0.008
Range	187–1363	107–1115	104–1188	99.1–1268	155–1445	
*Total water-soluble choline*						
Median (IQR)	1727 (366)	1219 (410)	1200 (308)	1289 (404)	1230 (344)	0.224
Range	955–2117	768–1817	809–1894	752–1896	652–2729	

^1^*n* = 20 mothers exclusively breastfeeding 2–6 months old infants. T1, milk collected before breakfast; T2, before lunch; T3, 45–60 min after lunch; T4, 45–60 min after dinner; T5, before bedtime. ^2^ *p* values for repeated-measures Friedman test for difference across time points within a woman.

## Supplementary Materials

The following are available online at https://www.mdpi.com/2072-6643/11/12/3024/s1: Supplementary Table S1: Storage duration and temperature condition for stability testing of human milk choline composition; Supplementary Table S2: Changes in water-soluble forms of choline concentrations in human milk under different storage conditions.
