# Supplementary material for: Variability of Water-Soluble Forms of Choline Concentrations in Human Milk during Storage, after Pasteurization, and among Women
Source: Nutrients. 2019 Dec 11;11(12):3024. doi: 10.3390/nu11123024 (PMC6949891; doi:10.3390/nu11123024)
Supplement: Supplementary file 1 [file nutrients-11-03024-s001.pdf]

Supplementary Table S1. Storage duration and temperature condition for stability testing of human milk choline composition

	5 min (baseline)	30 min	60 min	90 min	2 h	3 h	4 h	1 d	1 wk	6 mo
Fresh sample[1]										
Room temperature (23°C)										
Fridge (4°C)										
Freezing at -20°C (home and milk bank setting)										
Freezing at -80°C (laboratory setting)										

[1]Fresh sample analyzed within 5-10 min of pumping.

Supplementary Table S2. Changes in water-soluble forms of choline concentrations in human milk under different storage conditions[1]

Time	Room temperature (*n*=6)		4°C (*n*=5)		-20°C (*n*=5)		-80°C (*n*=5)	
Unit	µmol/L	*%change from baseline*	µmol/L	*%change from baseline*	µmol/L	*%change from baseline*	µmol/L	*%change from baseline*
Total water-soluble choline								
Baseline [2]	1231 ± 305		1157 ± 275		1157 ± 275		1157 ± 275	
30 min	1037 ± 258	-13 ± 22 (-51, 11)						
60 min	1104 ± 260	-10 ± 9 (-21, 3.2)	-		-	-	-	-
90 min	1057 ± 222	-13 ± 15 (-31, 7.7)	-		-	-	-	-
120 min	1059 ± 291	-14 ± 14 (-30, 12)	-		-	-	-	-
3h	1116 ± 200	-8 ± 10 (-18, 6.0)	-		-	-	-	-
4h	1217 ± 194	1 ± 17 (-19, 22)	-		-	-	-	-
24h	-	-	1087 ± 155	-3.5 ± 20 (-25, 24)	1081 ± 154	-3.2 ± 22 (-38, 17)	1118 ± 235	-1.0 ± 25 (-26, 30)
1w	-	-	-	-	1455 ± 504	37 ± 34 (-8.4, 66)	1361 ± 431	29 ± 33 (-14, 65)
6 mo	-	-	-	-	-	-	1419 ± 129	32 ± 6.7 (24, 36)
Free choline concentrations								
Baseline [2]	122 ± 38		128 ± 40		128 ± 40		128 ± 40	
30 min	106 ± 55	8 ± 41 (-87, 31)						
60 min	121 ± 36	1 ± 15 (-18, 24)	-		-	-	-	-
90 min	138 ± 49	12 ± 13 (-10, 27)	-		-	-	-	-
120 min	158 ± 55	29 ± 18 (0, 51)	-		-	-	-	-
3h	177 ± 64 [3]	44 ± 22 (8, 66)	-		-	-	-	-
4h	190 ± 75 [3]	53 ± 18 (31, 79)	-		-	-	-	-
24h	-	-	182 ± 115	35 ± 57 (-31, 95)	135 ± 67	2 ± 30 (-48,30)	101 ± 64	-23 ± 33 (-58, 16)
1w	-	-	-	-	175 ± 48	38 ± 64 (-23, 126)	131 ± 31	-1 ±27 (-27, 37)
6 mo	-	-	-	-	-	-	188 ± 64 [3]	56 ± 36 (3, 106)

[1] Data are presented as mean ± SD and %change as mean ± SD (minimum, maximum) compared to baseline concentrations in a complete human milk expression. [2] Baseline refers to the analysis completed right after aliquoting out samples, and thus prior to any storage. [3] Significantly different compared to baseline concentrations (Wilcoxon signed-rank test, $P<0.05$).

Supplementary Table S2 (continued). Changes in water-soluble forms of choline concentrations in human milk under different storage conditions[1]

**Time**	**Room temperature** (*n*=6)		**4°C** (*n*=5)		**-20°C** (*n*=5)		**-80°C** (*n*=5)	
Unit	µmol/L	*%change from baseline*	µmol/L	*%change from baseline*	µmol/L	*%change from baseline*	µmol/L	*%change from baseline*
Phosphocholine concentrations								
Baseline [2]	562 ± 225		483 ± 128		483 ± 128		483 ± 128	
30 min	449 ± 191	-16 ± 28 (-65, 17)						
60 min	445 ± 191	-19 ± 22 (-46, 16)	-		-	-	-	-
90 min	395 ± 148	-27 ± 23 (-48, 2)	-		-	-	-	-
120 min	386 ± 219	-33 ± 21 (-47, 6)	-		-	-	-	-
3h	399 ± 169	-27 ± 20 (-50, 2)	-		-	-	-	-
4h	468 ± 133	-9 ± 32 (-45, 38)	-		-	-	-	-
24h	-	-	320 ± 107	-30 +28 (-55, 8)	376 ± 127	-16 ± 38 (-59, 40)	415 ± 150	-7 ± 49 (-51,70)
1w	-	-	-	-	471 ± 200	7 ± 31 (-27, 43)	489 ± 228	9 ± 31 (-14, 53)
6 mo	-	-	-	-	-	-	582 ± 261	9 ± 25 (-18, 32)
Glycerophosphocholine concentrations								
Baseline [2]	547 ± 221		547 ± 247		547 ± 247		547 ± 247	
30 min	482 ± 170	-7 ± 23 (-47, 18)						
60 min	539 ± 245	-2 ± 11 (-16, 11)	-		-	-	-	-
90 min	524 ± 248	-5 ± 14 (-27, 13)	-		-	-	-	-
120 min	516 ± 252	-6 ± 20 (-40, 14)	-		-	-	-	-
3h	540 ± 206	0 ± 10 (-13, 13)	-		-	-	-	-
4h	559 ± 209	4 ± 13 (-13, 26)	-		-	-	-	-
24h	-	-	585 ± 221	10 ± 9 (-3, 20)	569 ± 210	10 ± 22 (-21, 34)	601 ± 241	12 ± 13 (-4, 30)
1w	-	-	-	-	809 ± 516	72 ± 66 (-24, 125)	741 ± 419	63 ± 67 (-33, 121)
6 mo	-	-	-	-	-		853 ± 267 [3]	68 ± 62 (31, 190)

[1] Data are presented as mean ± SD and %change as mean ± SD (minimum, maximum) compared to baseline concentrations in a complete human milk expression. [2] Baseline refers to the analysis completed right after aliquoting out samples, and thus prior to any storage. [3] Significantly different compared to baseline concentrations (Wilcoxon signed-rank test, $P<0.05$).
